# Supplementary material for: A meaningful prediction of functional decline in amyotrophic lateral sclerosis based on multi-event survival analysis
Source: PLoS One. 2025 Nov 18;20(11):e0336476. doi: 10.1371/journal.pone.0336476 (PMC12626301; doi:10.1371/journal.pone.0336476)
Supplement: S1 Table — (DOCX) [file pone.0336476.s001.docx]

PRO-ACT Covariates (d=8)

| Covariate |
| --- |
| ALSFRS_R_Total |
| Age |
| Sex |
| Site_of_Onset |
| Onset_Delta |
| DiseaseProgressionRate |
| Subject_used_Riluzole |
| FVC_Mean |
