# Supplementary material for: A meaningful prediction of functional decline in amyotrophic lateral sclerosis based on multi-event survival analysis
Source: PLoS One. 2025 Nov 18;20(11):e0336476. doi: 10.1371/journal.pone.0336476 (PMC12626301; doi:10.1371/journal.pone.0336476)
Supplement: S2 Table — (DOCX) [file pone.0336476.s002.docx]

PRO-ACT Missing Data (N=3220)

| Covariate | Missing Rows |
| --- | --- |
| ALSFRS_R_Total | 901 |
| Age | 212 |
| Site_of_Onset | 1267 |
| DiseaseProgressionRate | 901 |
| Subject_used_Riluzole | 572 |
| FVC_Mean | 798 |
