# Supplementary material for: A meaningful prediction of functional decline in amyotrophic lateral sclerosis based on multi-event survival analysis
Source: PLoS One. 2025 Nov 18;20(11):e0336476. doi: 10.1371/journal.pone.0336476 (PMC12626301; doi:10.1371/journal.pone.0336476)
Supplement: S3 Table — (DOCX) [file pone.0336476.s003.docx]

CoxPH Hyperparameters

| Parameter | Value |
| --- | --- |
| alpha | 0.001 |
| ties | breslow |
| n_iter | 100 |
| tol | 1e-09 |

RSF Hyperparameters

| Parameter | Value |
| --- | --- |
| n_estimators | 100 |
| max_depth | 3 |
| min_samples_split | 60 |
| min_samples_leaf | 30 |
| max_features | None |
| random_state | 0 |

DeepSurv Hyperparameters

| Parameter | Value |
| --- | --- |
| hidden_size | 32 |
| verbose | False |
| lr | 0.001 |
| c1 | 0.01 |
| num_epochs | 1000 |
| dropout | 0.25 |
| batch_size | 32 |
| early_stop | True |
| patience | 10 |

MTLR Hyperparameters

| Parameter | Value |
| --- | --- |
| hidden_size | 32 |
| verbose | False |
| lr | 0.001 |
| c1 | 0.01 |
| num_epochs | 1000 |
| dropout | 0.25 |
| batch_size | 32 |
| early_stop | True |
| patience | 10 |

MENSA Hyperparameters

| Parameter | Value |
| --- | --- |
| layers | [32] |
| lr | 0.001 |
| n_epochs | 1000 |
| n_dists | 3 |
| batch_size | 32 |
