# Supplementary material for: A meaningful prediction of functional decline in amyotrophic lateral sclerosis based on multi-event survival analysis
Source: PLoS One. 2025 Nov 18;20(11):e0336476. doi: 10.1371/journal.pone.0336476 (PMC12626301; doi:10.1371/journal.pone.0336476)
Supplement: S4 Table — (DOCX) [file pone.0336476.s004.docx]

Mr. Smith Covariates

| Covariate | Value |
| --- | --- |
| Onset_Delta | 545 |
| ALSFRS_R_Total | 37 |
| Age | 72 |
| Sex | Male |
| Site_of_Onset | Limb |
| DiseaseProgressionRate | 0.605505 |
| Subject_used_Riluzole | No |
| FVC_Mean | 2.69 |
